# The chemokine CX3CL1 promotes intraperitoneal tumour growth despite enhanced T-cell recruitment in ovarian cancer

**DOI:** 10.1016/j.neo.2025.101130

**Published:** 2025-01-24

**Authors:** Stefanie Seitz, Tobias F. Dreyer, Christoph Stange, Katja Steiger, Dirk Wohlleber, Martina Anton, Thuý An Pham, Dominique Sauter-Peschke, Ute Reuning, Gabriele Multhoff, Wilko Weichert, Marion Kiechle, Viktor Magdolen, Holger Bronger

**Affiliations:** a Department of Gynecology and Obstetrics, Technical University of Munich, 81675 Munich, Germany; b Comparative Experimental Pathology, Institute of Pathology, Technical University of Munich, 81675 Munich, Germany; c Institute of Molecular Immunology, Klinikum rechts der Isar, Technical University of Munich, 81675 Munich, Germany; d Department of Radiation Oncology, Technical University of Munich, TranslaTUM, 81675 Munich, Germany; e Institute of Pathology, Technical University of Munich, 81675 Munich, Germany; f German Cancer Consortium (DKTK), partner site Munich, and German Cancer Research Center (DKFZ), Heidelberg, Germany

**Keywords:** Chemokines, CX3CL1, Tumour-infiltrating lymphocytes, PARP inhibition, Mouse model

## Abstract

T-cell recruiting chemokines are required for a successful immune intervention in ovarian cancer, and also for the efficacy of modern anticancer agents such as PARP inhibitors. The chemokine CX3CL1 recruits tumour-suppressive T-cells into solid tumours, but also mediates cell–cell adhesions, e.g. of tumour cells, through its membrane-bound form. So far, its role in ovarian cancer has only been rudimentarily addressed. We show that high CX3CL1 expression significantly correlates with worsened survival in human high-grade serous ovarian cancer (n=219). In preclinical ovarian cancer, CX3CL1 plays a dual role, as it enhances the adaptive anti-tumour response, but overall still promotes tumour growth, the latter as a feature of the intraperitoneal environment. Moreover, PARP inhibitors are able to increase CX3CL1 release from human ovarian cancer cells. Collectively, our study shows that CX3CL1 is a driver of intraperitoneal tumour growth in ovarian cancer, a feature that may compromise the anticancer effect of CX3CL1-inducing PARP inhibitors.

## Introduction

Ovarian cancer is the fifth leading cause of death in women in the Western world, and survival remains unsatisfactorily low despite numerous advances in systemic therapy. Pillars of therapy for ovarian carcinoma are radical surgery and chemotherapy, supplemented by antiangiogenic agents when appropriate. In recent years, inhibitors of poly(ADP-ribose) polymerase 1 (PARP1) have revolutionised the therapy of ovarian cancer, particularly in tumours that have preexisting damage to their homologous recombination [1]. In this context, PARP inhibitors act by interfering with DNA repair, in which PARP1 is involved. Another mechanism of action is based on the activation of the cGAS/STING pathway through the accumulation of cytosolic DNA fragments in the tumour cell, which ultimately leads to an interferon-like immune response, in particular through the secretion of T-cell recruiting chemokines such as CCL5 or CXCL10 [2]. This latter mechanism of action may also explain why immune checkpoint inhibitors (e.g. against the PD-1/PD-L1 axis), which alone show no effect in ovarian cancer, act synergistically in combination with PARP inhibitors [3]. This synergism appears to be functionally dependent on the action of chemokines such as CXCL10, which promote a functional anti-tumour immune response through T-cell recruitment and activation [[4], [5], [6], [7]].

CX3CL1 (fractalkine) is another chemokine that is also involved in T-cell recruitment to solid tumours [8]. In addition to its soluble, chemotactically active form, it is initially produced as a membrane-bound protein that is particularly involved in cell-cell adhesion processes [9]. Its function in ovarian cancer has been much less studied than that of CXCL9 or CXCL10. Previous work has mainly shown that the receptor for CX3CL1, CX3CR1, may promote proliferation, migration, and adhesion of ovarian cancer cells and thus promote tumour spread [10,11]. This would be in diametric opposition to its T-cell recruiting property.

The present study aimed at further elucidating the function of CX3CL1 in high-grade serous ovarian carcinoma (HGSOC). In contrast to CXCL9 or CXCL10, and also in contrast to its role in other carcinomas such as breast cancer, CX3CL1 overexpression was found to represent an independent negative prognostic marker in human HGSOC. Despite increased tumour-suppressive activation of the adaptive immune response, its overexpression in a syngeneic orthotopic ovarian carcinoma mouse model resulted in accelerated tumour growth. This indeed seems to be a peculiarity of the intraperitoneal milieu, as CX3CL1 overexpression in subcutaneous tumour implantation of the same cell line resulted in successful tumour suppression, which was most likely immune-mediated. Finally, we show that PARP inhibitors can induce CX3CL1 in ovarian cancer cells, which could compromise their therapeutic efficacy.

## Materials and methods

### Human tissue samples and patient characteristics

For immunohistochemical staining and retrospective survival analysis, we included formalin-fixed, paraffin-embedded tumour specimens from 219 high-grade serous ovarian cancer patients with FIGO stage III or IV (International Federation of Gynecology and Obstetrics) in our study. Written informed consent was obtained from all patients, which underwent surgery between 1990 and 2014 at the Technical University of Munich Hospital (Klinikum rechts der Isar, Department of Obstetrics and Gynecology). Detailed patient characteristics are given in N Metastatic tissue was available for IHC analysis in the omentum (122 cases) and in the peritoneum (126 cases).

### Immunohistochemistry on human samples

Formalin-fixed, paraffin-embedded (FFPE) tumour cores on Tissue Microarrays (TMAs) were cut in 3 µm sections. After deparaffination with xylene and a graded series of ethanol, endogenous peroxidase activity was quenched by H_2_O_2_ incubation (3 %, 20 min). TBS-T washing was performed between each protocol step. The primary monoclonal mouse CX3CL1 antibody (#MAB3651, R&D Systems) was applied on sections for 1 h at room temperature with a 1:100 dilution in antibody diluent (#ZUC025, Zytomed Systems). Zyto-Chem PLUS HRP One-Step Polymer anti-mouse/rabbit (#ZUC053, Zytomed Systems) and DAB Substrate Kit (#DAB530, Zytomed) were used according to the manufacturer's instructions to detect primary antibody binding. Sections were counterstained with hematoxylin and dehydrated with an ascending alcohol series. NanoZoomer Digital Pathology RS (Hamamatsu, Japan) was used to scan histological images for further CX3CL1 quantification analysis. RGB digital images were taken for each TMA tumour tissue dot applying the NDP.View2 software (version V2.8.24) and loaded into the ImageJ platform (version 1.8.0). Using the IHC Profiler plugin, the CX3CL1 antibody DAB signal was separated and the optical density was measured within three tumour areas of one TMA dot. In order to bring high scores in line with high staining intensities, final mean density values were subtracted from the maximum signal of 255. Finally, patients were assembled in low and high CX3CL1 expressing groups using a signal threshold of 75.

### Cell lines and cell culture

The murine ID8-*Trp53^−/−^* ovarian surface epithelial cell line was kindly provided by Prof. Ian McNeish's laboratory (University of Glasgow) [12]. The human packaging cell line HEK293T was obtained from DSMZ (Leibniz Institute). Human ovarian cancer cell lines CAOV-3 and OVCAR-3 were purchased from the American Type Culture Collection (ATCC), whereas human OV-MZ-6 ovarian cancer cells were obtained from Möbus et al. [13]. Cells were cultured in DMEM with 10% FCS and 10 mM HEPES (ID8-*Trp53^−/−^*, HEK293T, CAOV-3, OV-MZ6) or RPMI 1640 media with 20% FCS and 10 mM HEPES (OVCAR-3). As an additional supplement, 1% ITS-solution containing 10 μg/mL insulin, 5.5 μg/mL transferrin and 6.7 ng/mL sodium selenite was added for ID8-*Trp53^−/−^* media, whereby 0.1 µg/mL bovine pancreas insulin solution was included in OVCAR-3 media. The cells were grown in a humidified 5% CO_2_ atmosphere at 37 °C, subcultured with 0.05 % EDTA or 0.25 % trypsin solution and underwent subsequent mycoplasma testing.

### Generation of luciferase-expressing ID8*luc*-*Trp53^−/−^* cells

A PCR fragment containing the *eGFP*2A*luc* (“enhanced green fluorescent protein” – “2A self-cleaving peptide” – “click beetle luciferase”) sequence was incorporated at the Bam HI cleaving site of the pHIV-7 transfer vector with SF (“spleen-focus-forming virus”) promotor. Viral supernatants were generated via transient calcium-phosphate transfection of 293T cells with pHIV-7 SF *eGFP*2A*luc* transfer vector and three packaging vectors (pMD.GP, pRSV-rev and pHCMV.G). ID8-*Trp53^−/−^* cells were transduced at 37 °C with filtered lentiviral supernatant containing 8 µg/mL Polybrene. eGFP-positive cells were isolated via fluorescence-assisted cell sorting (FACS) and subcultured for subsequent experiments.

### Cx3cl1 overexpression in murine cell lines

Stable overexpression of murine Cx3cl1 in ID8-*Trp53^−/−^* or ID8*luc*-*Trp53^−/−^* cells was performed using the ViraSafe^TM^ Lentiviral Expression System (Neo) from Cell Biolabs (#VPK-213-ECO). cDNA derived from a Cx3cl1 mouse clone (#NM_009142, Origene) was ligated into the multiple cloning site of the pSMPUW-Neo transfer vector before the generation of viral supernatant by HEK293T cells. After viral transduction, G418 (1 mg/mL) was added to the cell culture medium to select ID8-*Trp53^−/−^* or ID8*luc*-*Trp53^−/−^* cells with incorporated pSMPUW-Neo-*Cx3cl1*. As a control, target cells were treated analogously with the pSMPUW-Neo-empty vector. After one week, single-cell clones were selected by limiting dilution and tested for Cx3cl1 expression with qPCR or with ELISA technique.

### Animal experiments

Projects were carried out following the institutional guidelines of the Preclinical Research Center at the Technical University of Munich. Approval for all experimental animal procedures was obtained from the Government of Upper Bavaria (Regierung von Oberbayern). Six- to eight-week-old female C57BL/6 (strain 632) and athymic nude mice (strain 490) were obtained from Charles River. Cell lines underwent mycoplasma testing before *in vivo* application. To evaluate the effect of intratumoural Cx3cl1 in a metastatic tumour model, 1×10^7^ ID8-*Trp53^−/−^*Control or ID8-*Trp53^−/−^Cx3cl1^+^* cells were dissolved in 250 µL PBS and intraperitoneally inoculated in C57BL/6 or athymic nude mice (n = 7 each group). Besides diffuse carcinomatosis throughout the peritoneal cavity, hemorrhagic ascites developed and visible abdominal swelling was defined as onset of ascites. Severe accumulation of ascitic fluid and/or poor health condition were predefined as finalization endpoints. For further chemokine analysis, ascitic fluid was collected and mesenteric tumour implants were partly snap-frozen and partly fixed in 4 % paraformaldehyde for paraffin-embedding. To examine the effect of Cx3cl1 in an artificial primary tumour model with subsequent *in vivo* bioluminescence imaging, 1×10^7^ ID8*luc*-*Trp53^−/−^*Control or ID8*luc*-*Trp53^−/−^Cx3cl1^+^* cells were dissolved in 250 µL PBS and subcutaneously inoculated in C57BL/6 mice (n = 6 each group). Injected mice developed a palpable tumour formation after approximately 100 days, which was documented in tumour diameter twice a week by bioluminescence measurement. Finalization criteria were defined by the tumour breaking through the skin layer, by reaching a tumour diameter of 1.5 cm and/or by a persistent poor health condition. Subcutaneous tumour material was partly snap-frozen for qPCR analysis and partly fixed in 4 % paraformaldehyde for immunohistochemical staining.

### Non-invasive *in vivo* bioluminescence imaging

Tumour growth of luciferase-expressing tumour cells was measured *in vivo* once per week via intraperitoneal administration of 150 µL D-luciferin substrate (50 mM, PJK Biotech). The mice were then placed in the Perkin Elmer IVIS Lumina LT-Series III In Vivo Imaging System under sustained anesthesia with a 2.5 % isoflurane inhalation system. Five minutes after D-luciferin substrate application, both a grayscale reference image and a bioluminescence image were acquired with a two-minute exposure time via the Living Image 4.0 Software from Perkin Elmer. For evaluation, a region of interest of equal size was placed on the abdomen of each mouse and the photons emitted by the luciferase-expressing tumour cells were measured as “flux” in photons/second.

### Immunohistochemistry on murine tissue

Immunohistochemical staining of the markers Ki67, F4/80, CD206 and CX3CR1 was performed with 3 µm tumour tissue sections using heat-induced epitope retrieval (citrate buffer pH 6), peroxide block (3 % H_2_O_2_) and Zyto-Chem Plus HRP One-Step Polymer System as mentioned above. Primary antibody dilutions were as follows: 1:1,000 for polyclonal rabbit anti-Ki67 (#NB500-170, Novus Biologicals); 1:300 for monoclonal rabbit anti-F4/80 (#70076, Cell Signaling Technology); 1:750 for polyclonal rabbit anti-CD206 (#18704-1-AP, Proteintech); and 1:750 for polyclonal rabbit anti-CX3CR1 (#ab8021, Abcam). Additional stainings (CD3, CD8, Granzyme B, Ly6G, Foxp3) were performed on an automated immunostainer (Bond Rxm, Leica Biosystems) with epitope retrieval (citrate buffer pH6 or EDTA buffer pH9) and following primary antibody dilutions: 1:100 for rabbit anti-CD3 (clone SP7, #CI597C01, DCS Diagnostics); 1:100 for monoclonal rat anti-CD8 (clone GHH8, #DIA-808, Dianova); 1:1,000 for polyclonal rabbit anti-Granzyme B (#ab4059, abcam); 1:1,000 for monoclonal rat anti-Ly6G (#NB500-170, Novus Biologicals); and 1:2,000 for polyclonal rabbit anti-Foxp3 (#ab4728, Abcam). Signal detection was achieved with the BOND Polymer Refine Detection Kit (#DS9800, Leica Biosystems) including hematoxylin for counterstaining. Stained slides were scanned using the slide scanner AT-2 (Leica Biosystems) and representative images were taken at 40-fold magnification in Aperio ImageScope software version 12.3 (Leica Biosystems). Intratumoural DAB-positive cells were manually counted within five report images (640×480 fixed region size pixel) per slide. QuPath open-source software version 0.2.3 24 was used to digitally evaluate DAB-positive cells as percent of all cells within five intratumoural regions of interest (ROIs) with a mean cell detection of 2000 cells each.

### *In vitro* stimulation experiments

In order to test the impact of PARP-inhibition on CX3CL1 chemokine secretion, human ovarian cancer cells (OV-MZ-6, CAOV-3 and OVCAR-3) were seeded at 60 % confluency, starved for 24 h and stimulated for 48 h with 10 µM olaparib or niraparib ± TNF-α (10 ng/mL). During stimulation, the cell culture medium was supplemented with 5% FCS. Dimethyl sulfoxide (DMSO) was used as a PARP-inhibitor solvent control. Soluble CX3CL1 antigen levels were determined by ELISA in cell supernatants. Another *in vitro* stimulation protocol was prosecuted to examine the effect of ADAM-17 or ADAM-10 inhibition on CX3CL1 shedding. Therefore, human cancer cell lines OV-MZ-6 and OVCAR-3, as well as murine ID8-*Trp53^−/−^* cells were seeded at 60 % confluency, starved for 24 h and stimulated for 24 h in starvation medium with the ADAM-17 inhibitor TAPI-2 (50 µM, #SML0420, Sigma-Aldrich) or the ADAM-10 inhibitor GI254023X (5 µM, # 3995, Bio-Techne) under the addition of 50 ng/ml TNF-α or correspondent solving control.

### Statistics

Statistical analysis was performed with SPSS Statistics (V26) and GraphPad Prism Software (version 9.0.1). ANOVA testing evaluated significant differences between groups. Kaplan–Meier plots were compared by long-rank testing. Univariate and multivariate analyses were performed via Cox-regression. *P* values < 0.05 were considered significant. Log-rank analysis was validated via bootstrapping with 10,000 combinations (data not shown). Bars and horizontal lines represent the mean ± standard error of the mean (SEM). Within dot plots, each dot represents an individual mouse. *In vitro* experiments were performed independently for three times.

## Results

### CX3CL1 overexpression is an independent prognostic marker for reduced progression-free and overall survival in advanced high-grade serous ovarian cancer (HGSOC)

**Fig. 1:**
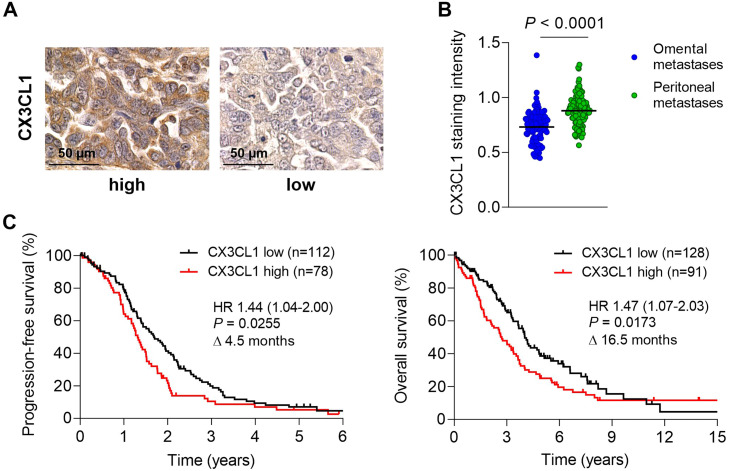
CX3CL1 is an unfavourable prognostic marker in high-grade serous ovarian cancer (HGSOC) patients. A, Representative immunohistochemistry (IHC) images of high or low CX3CL1 expression in a patient cohort of high-grade serous ovarian cancer (scale bars represent 50 µm). **B,** Digitally analysed and quantified CX3CL1 IHC staining intensity in omental (n = 122) and peritoneal (n = 126) metastases (mean values were normalised to TMA control tissue). **C,** Kaplan Meier curves showing progression free survival (left) and overall survival (right) in CX3CL1 high vs. low expressing groups of HGSOC.

To explore a possible prognostic impact of CX3CL1 in advanced ovarian cancer, a cohort of 219 HGSOC primary (ovarian) tumours (FIGO III and IV only) was immunohistochemically stained against CX3CL1 and semiquantitatively scored and confirmed by an experienced pathologist (KS) (Fig. 1A and Table S1). CX3CL1 was mainly localised in tumour cells. CX3CL1 expression was also retained in intraabdominal metastases, with digital analysis showing significantly higher CX3CL1 staining intensities in metastases of the parietal peritoneum than in omental metastases (*P* < 0.0001, Fig. 1B). Patients with CX3CL1 overexpressing tumours showed a significantly shorter progression-free survival (PFS) than the low-expressing group (median 15.5 vs. 20 months; HR 1.44, 95% CI 1.04-2.00; *P =* 0.026; Fig. 1C). This survival disadvantage of the CX3CL1^high^ group increased to 16.5 months in overall survival (median 31.5 (CX3CL1^high^) vs. 48 months (CX3CL1^low^); HR 1.47, 95 % CI 1.07-2.03; *P =* 0.0173; Fig. 1C). In addition, univariate COX regression analyses confirmed FIGO stage, post-surgical tumour burden, ascites volume, and nodal status as predictors for PFS and OS in our cohort (Table S2). In multivariate analysis, CX3CL1 remained an independent prognostic marker for poor outcome both for PFS (HR 2.07, 95 % CI 1.23-3.50; *P =* 0.006) and OS (HR 1.83, 95 % CI 1.14-2.93; *P =* 0.012; Table 1). The classic clinicopathologic factors post-surgical tumour, ascites and FIGO stage remained prognostic in multivariate analysis as well (Table 1).

**Table 1:** Multivariate Cox regression analysis of clinical outcome in high-grade serous ovarian cancer patients (FIGO III/IV) with respect to clinical parameters and CX3CL1 expression.

Clinical parameters	N	PFS	*P*	n	OS	*P*
		HR (95% CI)			HR (95% CI)	
**Age (years)**			0.866			0.807
≤ 60	45	1		47	1	
> 60	40	0.96 (0.57–1.61)		48	1.06 (0.66–1.70)	
**FIGO stage**			**0.052**			**0.001**
III	58	1		63	1	
IV	27	1.77 (0.99–3.14)		32	2.30 (1.40–3.75)	
**Residual tumour mass**			**0.072**			**<0.001**
0 cm	36	1		39	1	
> 0 cm	49	1.66 (0.96–2.88)		56	2.81 (1.61–4.90)	
**Ascites volume**			**0.003**			0.083
≤ 500 ml	46	1		51	1	
> 500 ml	39	2.34 (1.34–4.10)		44	1.58 (0.94–2.65)	
**Nodal status**			0.486			0.667
negative	26	1		27	1	
positive	59	0.82 (0.47–1.430)		68	0.89 (0.51–1.54)	
**CX3CL1 expression**			**0.006**			**0.012**
low	36	1		38	1	
high	49	2.07 (1.23–3.50)		57	1.83 (1.14–2.93)	

**CI**, confidence interval; **FIGO**, International Federation of Gynecology and Obstetrics; **HR**, hazard ratio; **OS**, overall survival; **PFS**, progression-free survival. Significant values (*P* < 0.05) are indicated in bold.

Thus, in contrast to its favourable prognostic role in other cancer entities and in contrast to the positive prognostic impact of other T-cell and NK cell recruiting chemokines (e.g. CXCL9 and CXCL10) in ovarian cancer, elevated CX3CL1 levels are associated with worsened outcome in HGSOC. Next, we wanted to unravel a functional basis for this observation.

### CX3CL1 is expressed by ovarian cancer cells *in vitro* both as a membrane-bound form and a soluble chemokine

Having detected CX3CL1 expression by immunohistochemistry in tumour cells from HGSOC patients, we next sought to confirm its expression and also the regulation of CX3CL1 in ovarian cancer cells *in vitro*. To this end, the membrane-bound form was determined by FACS analysis and immunocytochemical staining of living cells, and the secreted form was determined by ELISA from cell supernatants. Furthermore, we wanted to know whether, in ovarian cancer cells, the transition of these forms is controlled by proteases, i.e. ADAM10 or ADAM17, which had been associated with CX3CL1 cleavage in other cell systems before.

**Fig. 2:**
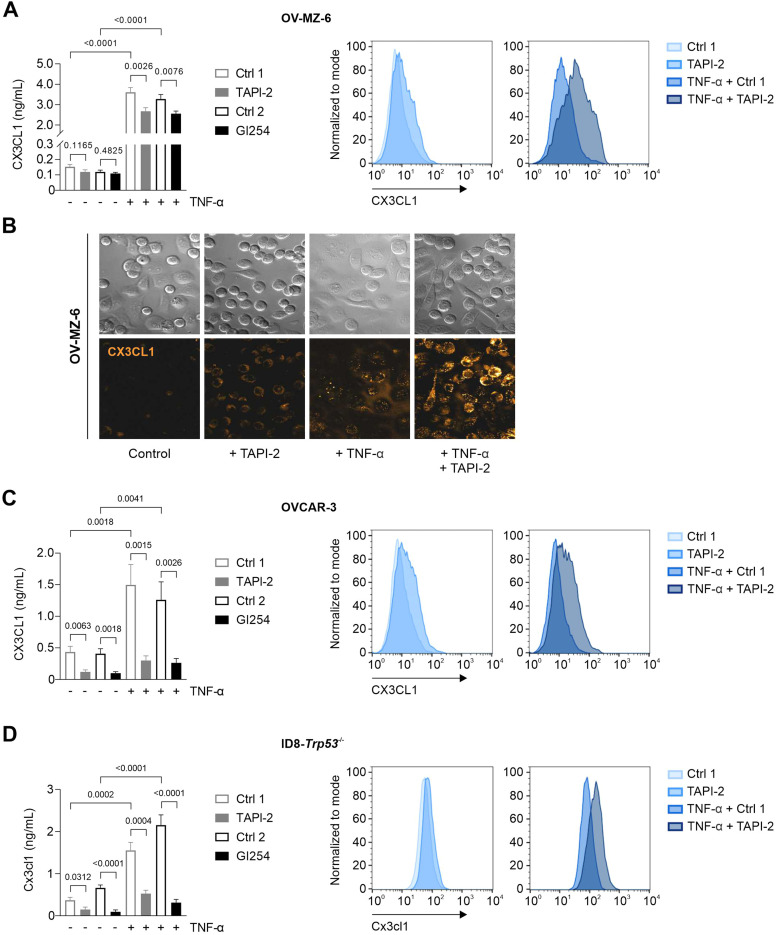
CX3CL1 expression and regulation in human and murine ovarian cancer cell lines. OV-MZ-6 cells **(A, B)**, OVCAR-3 cells **(C)**, or ID8-*Trp53^−/−^* cells **(D)** were stimulated for 24 h ± TNF-α (50 ng/mL) and ± TAPI-2 (50 µM) or ± GI254 (5 µM) or the corresponding solvent controls as indicated. Bar graphs on the left show soluble CX3CL1 in cell supernatant measured after 24 h by ELISA (mean ± SEM). The plots on the right show membrane-bound CX3CL1 as determined via FACS analysis. **B,** Immunocytochemical staining of CX3CL1 in OV-MZ-6 cells stimulated with or without TNF-α in the presence or absence of TAPI-2, visualised by confocal laser scanning microscopy (CLSM) using an Alexa 488-conjugated secondary antibody.

OV-MZ-6 cells express basal CX3CL1 at very low concentrations, and ADAM10 inhibiton by GI254 or ADAM17 inhibition by TAPI-2 did not significantly reduce its secretion (Fig. 2A). Incubation of the cells with TNF-α for 24 h strongly induced both its secretion and the membrane-bound fraction (Fig. 2A, secreted CX3CL1 elevated from 0.15 ng/mL to 3.6 ng/mL, *P* < 0.0001). Compared to the corresponding solvent controls, soluble CX3CL1 was diminished from 3.6 to 2.7 ng/mL under ADAM17 inhibition with TAPI-2 (*P* = 0.0026), and from 3.3 to 2.6 ng/mL under ADAM10 inhibition with GI254 (p = 0.0076, Fig. 2A). Correspondingly, FACS-analysis revealed an upregulation of membrane-bound CX3CL1 when cells were stimulated with TAPI-2 with or without the addition of TNF-α (Figg. 2A, S1A). This increase in membrane-bound CX3CL1 upon ADAM inhibition could also be replicated immunocytochemically (Fig. 2B).

Stimulation of human OVCAR-3 cells revealed highly significant effects of ADAM inhibition on CX3CL1 secretion, as basal values were reduced 3.6-fold by TAPI-2 (*P =* 0.0063) and 4-fold by GI254 (*P =* 0.0018, Fig. 2C). Upon TNF-α stimulation, effects were increased 5-fold and 4.8-fold, respectively (*P =* 0.0015 and *P =* 0.0026, Fig. 2C). FACS-analysis again revealed a clear right shift of normalised CX3CL1 fluorescence under TAPI-2 stimulation in OVCAR-3 cells (Fig. 2C).

These effects could also be reproduced in murine ID8-*Trp53^−/−^* cells, which were used for *in vivo* studies: TAPI-2 significantly reduced soluble murine Cx3cl1 by 2.5-fold under basal condition (*P =* 0.0312) and by 3-fold under TNF-α stimulation (*P <* 0.0001). GI254 diminished Cx3cl1 secretion 7-fold under both basal condition and TNF-α stimulation (*P =* 0.0004 and *P =* 0.0001, Fig. 2D). Corresponding elevation of the membrane-bound fraction of murine Cx3cl1 could be seen via FACS-measurements (Fig. 2D). In all three cell lines, the ADAM inhibitors had no effect on CXCL10 secretion, ruling out any off-target effects (Fig. S1B).

In summary, both membrane-bound and soluble isoforms of CX3CL1 are expressed by human and murine ovarian carcinoma cells. They are inducible by inflammatory cytokines (e.g. TNF-α) and regulated by proteolytic cleavage.

### In a syngeneic mouse model of ovarian cancer, Cx3cl1 promotes tumour growth despite enhanced T-cell infiltration

**Fig. 3:**
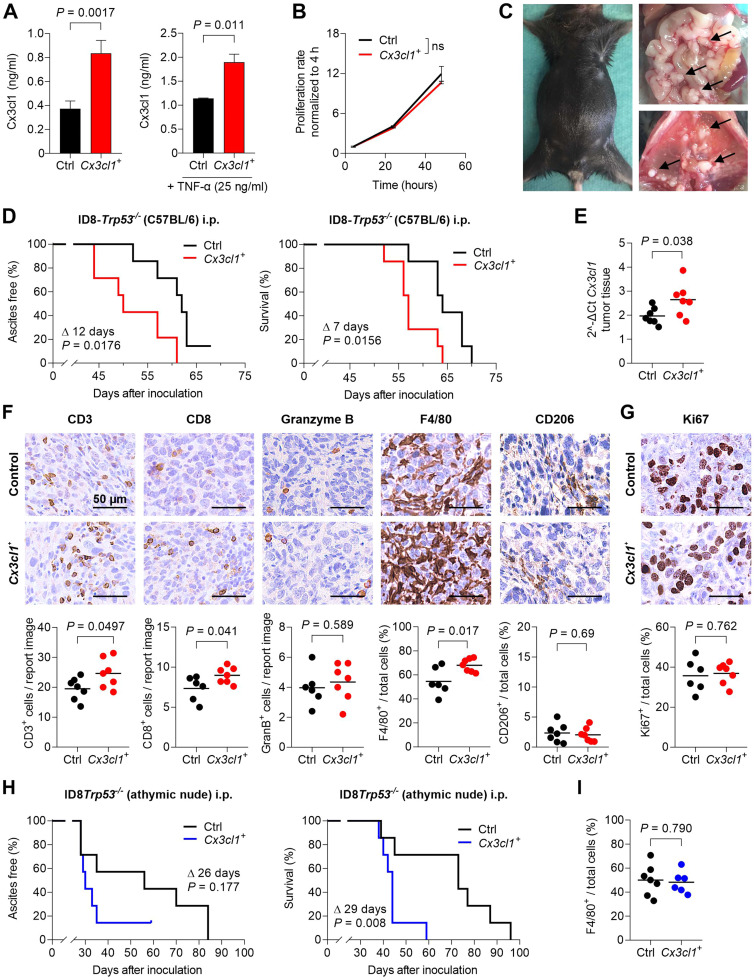
Cx3cl1 accelerates intraperitoneal tumour growth while enhancing immune infiltration. A, Expression of murine Cx3cl1 was verified in ID8-*Trp53^-/-^empty vector* (Ctrl) and ID8-*Trp53^-/-^Cx3cl1^+^* (*Cx3cl1^+^*) cells via cell supernatant ELISA measurement of untreated or TNF-α stimulated cells (24 h, 25 ng/mL). Bars represent mean ± SEM. **B,** Proliferation rates of ID8-*Trp53^-/-^*Ctrl and *Cx3cl1^+^* cells were measured via MTT assay and normalised to the baseline value at 4 h. **C**, Representative pictures of ascites accumulation (left) as well as tumour load on the mesentery (upper right) and diaphragm (lower right) at the time of finalization (black arrows indicate exemplary tumour nodes). **D,** Kaplan–Meier curves showing ascites-free (left) and overall survival (right) of C57BL/6 mice intraperitoneally injected with 1×10^7^ ID8-*Trp53^-/-^*Ctrl or *Cx3cl1^+^* cells (differences in median survival are indicated as Δ days). **E,** Relative *Cx3cl1* mRNA expression in mesenteric tumour tissue of ID8-*Trp53^-/-^*Ctrl or *Cx3cl1^+^* tumours at the end of the experiment. **F,** Quantification of tumour-infiltrating immune cell subsets (CD3, CD8, granzyme B, F4/80, CD206) by immunohistochemical stainings of ID8-*Trp53^-/-^*Ctrl or *Cx3cl1^+^* mesenteric tumour tissues. DAB positive immune cell markers (CD3, CD8, granzyme B) were manually counted in intratumoural report images. Positive macrophage subset cells (F4/80, CD206) were digitally evaluated as percentage of all cells. **G,** Ki67-positive proliferation status was digitally assessed as percentage of total cells. **H,** 1×10^7^ ID8-*Trp53^−/−^*Ctrl or *Cx3cl1^+^* cells were implanted intraperitoneally into athymic nude mice, and onset of ascites (left) and overall survival (right) was determined and shown as Kaplan–Meier curves (differences in median survival are indicated as Δ days). **I,** F4/80 macrophage marker was immunohistochemically stained in mesenteric tumour tissue from athymic nude mice, and positive cells were calculated as percentage of all cells. Horizontal lines in **E-G** and **I** indicate the mean, each dot indicates data from one individual mouse. Mouse experiments were conducted with at least 7 mice per group.

To explore the functional role of CX3CL1 in advanced ovarian cancer, we generated ID8-*Trp53^-/-^* cells overexpressing Cx3cl1 by lentiviral transduction. After 24 h, conditioned supernatants of ID8-*Trp53^-/-^Cx3cl1^+^* cells (referred to as ‘*Cx3cl1^+^*’) contained approx. 2.3-fold more soluble Cx3cl1 than that of the respective empty vector ID8-*Trp53^-/-^* cells (referred to as ‘Control’ or ‘Ctrl’; 0.84 vs. 0.37 ng/mL, *P =* 0.0017; Fig. 3A). After stimulation of both cell lines with TNF-α (25 ng/mL, 24 h), *Cx3cl1^+^* cells still secreted 1.7-fold more Cx3cl1 (*P =* 0.0011; Fig. 3A). This elevated *Cx3cl1* expression was also reproducible on the mRNA level (2.1-fold increase, *P =* 0.0028; Fig. S2A). Cx3cl1 overexpression, however, had no impact on tumour cell proliferation *in vitro* (Fig. 3B).

Next, 1×10^7^ Ctrl or *Cx3cl1^+^* cells were intraperitoneally implanted into C57BL/6 mice in which they mimicked the metastatic pattern of advanced human ovarian cancer (ascites formation, tumour outgrow in the mesentery and the diaphragm; Fig. 3C). Cx3cl1 overexpression significantly accelerated formation of ascites (50 vs. 62 days, *P =* 0.0176; Fig. 3D) and, more importantly, led to a meaningful shortening of survival in these mice (57 vs. 64 days, *P =* 0.016; Fig. 3D). After finalization due to reached endpoints, *Cx3cl1* mRNA expression in mesenteric tumour tissue was still significantly higher in the *Cx3cl1* overexpressing group (2.64 vs. 1.97 2^^-ΔCt^, *P =* 0.038; Fig. 3E). However, no increased CX3CL1 concentration was detectable in ascites of mice with *Cx3cl1^+^* tumours (0.08 vs. 0.10 ng/mg, *P =* 0.72; Fig. S2B).

As the CX3CL1 chemokine is expected to promote the recruitment of T-cells, natural killer cells and monocytes to the tumour microenvironment, we conducted immunohistochemical stainings of immune cell subsets. Compared to the ID8-*Trp53^-/-^* control group, *Cx3cl1^+^* tumours contained approximately 30 % more intratumoural CD3-positive (*P =* 0.050) and CD8-positive T-cells (*P =* 0.040), whereas no difference was seen in the amount of intratumoural Granzyme-B expressing cells (*P =* 0.589; Fig. 3F). As we had previously confirmed in preclinical breast cancer models [14], Cx3cl1 also attracted more F4/80-positive macrophages to the tumour microenvironment in the ID8-*Trp53^-/-^* model (median 68 % (Cx3cl1^+^) vs. 55 % (Control) of total cells, *P =* 0.017; Fig. 3F). However, we did not detect an increase in CD206-positive M2 macrophages which made up only a minor proportion of the entire macrophage population (median 2.0% vs 2.3 % of total cells, *P =* 0.69). Also, there was no difference in the infiltration of myeloid suppressor cells, as demonstrated via Ly6G immunohistochemistry (median 1.2 % vs. 1.3 % of total cells, *P =* 0.91, Fig. S2C). In accordance with our *in vitro* data (Fig. 3B), enhanced proliferation did not seem to account for the accelerated tumour outgrowth upon *Cx3cl1* overexpression, as Ki67-positivity was unaltered in *Cx3cl1^+^* tumours (median 37 % vs 36 % of total cells; *P =* 0.76; Fig. 3G). Of note, the receptor for the Cx3cl1 chemokine, Cx3cr1, that has been suggested to play a supportive role in ovarian cancer metastasis formation [11], was not found to be differentially expressed in *Cx3cl1^+^* or control tumours (Fig. S2D).

To further unravel the contradictory result of an increased tumour-suppressive T-cell infiltration and shortened survival upon Cx3cl1 overexpression, we repeated the experiment in female athymic nude mice and intraperitoneally inoculated 1×10^7^ ID8-*Trp53^-/-^* control or *Cx3cl1^+^* cells. Underlining the tumour-suppressing role of infiltrating T-cells, the difference in median overall survival significantly expanded from 7 days in immunocompetent (Fig. 3D) to 29 days in athymic nude mice (44 days (*Cx3cl1^+^*) vs. 73 days (Ctrl), *P =* 0.008; Fig. 3H). Also, the difference in time to onset of ascites extended as well from 12 days (Fig. 3D) to 26 days in the immunocompromised model (Fig. 3H), even if it failed to reach statistical significance here (30 vs. 56 days, *P =* 0.18). Interestingly, in this immunocompromised mouse model, we revealed a significantly higher Cx3cr1 expression in mesenteric tumour tissue of the ID8-*Trp53^-/-^Cx3cl1^+^* group (0.35 vs. 0.45 DAB intensity, *P =* 0.009, Fig. S2E). In contrast to the findings of the immunocompetent C57BL/6 model, the significantly higher macrophage infiltration within the ID8-*Trp53^-/-^Cx3cl1^+^* group was abrogated in athymic nude mice (48% vs. 50% of total cells, *P =* 0.79, Fig. 3I).

Thus, despite activating the adaptive immune system against ovarian cancer growth, most likely through T-cell recruitment, CX3CL1 overexpression promotes intraperitoneal tumour spread and shortens survival, very well reflecting the adverse prognostic impact in human HGSOC as shown before.

### In contrast to the intraperitoneal (orthotopic) ovarian cancer model, Cx3cl1 delays tumour growth when tumour cells are implanted subcutaneously

**Fig. 4:**
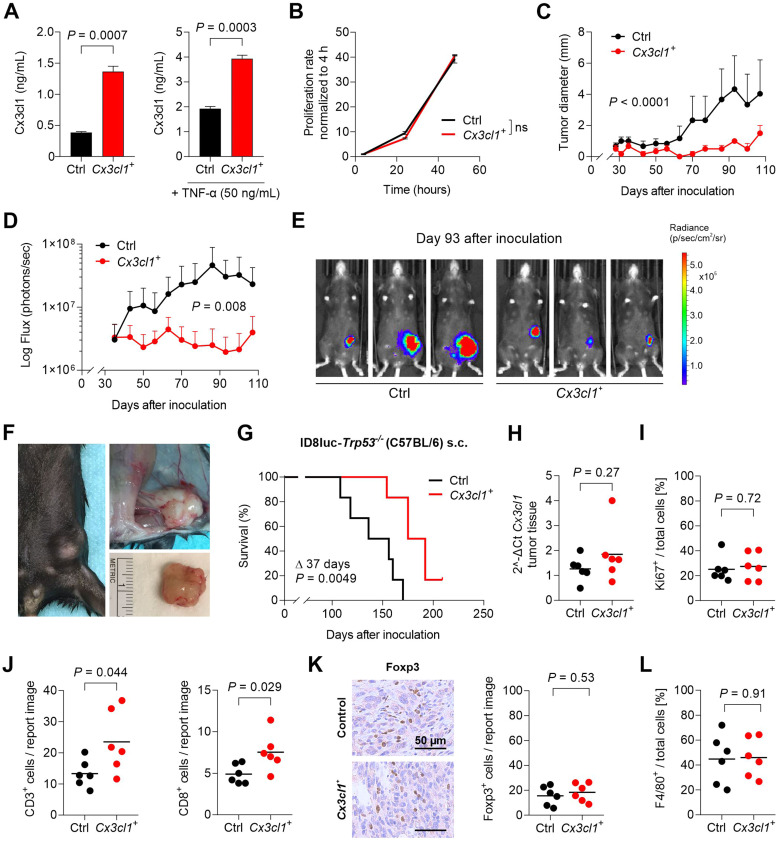
Cx3cl1 inhibits tumour growth in the subcutaneous ID8 ovarian cancer model. A, ID8*luc*-*Trp53^-/-^*Control (Ctrl) and ID8*luc*-*Trp53^-/-^Cx3cl1^+^* (*Cx3cl1^+^*) cells were tested for Cx3cl1 protein secretion into the cell supernatant of untreated or TNF-α (24 h, 50 ng/mL) stimulated cells by ELISA. Bars represent mean ± SEM. **B,** Cell proliferation rates of ID8*luc*-*Trp53^-/-^*Ctrl and *Cx3cl1^+^* cells were compared via manual counting with a Neubauer counting chamber and normalised to the baseline value at 4 h. **C-G,** 1×10^7^ ID8*luc*-*Trp53^-/-^*Ctrl or *Cx3cl1^+^* cells were subcutaneously inoculated into the flanks of C57BL/6 mice (n = 6 each group). **C,** Graph shows caliper measurement of maximal tumour diameter (mm) once a week until the first mouse reached predefined endpoints. **D,** Weekly flux (photons/sec) measurement of luciferase expressing tumour cells upon intraperitoneal luciferin substrate injection. **E,** Representative pictures of each group indicate bioluminescence signals (day 93). The pseudocolors represent the average radiance in the unit of p/s/cm^2^/sr with a maximum radiance of 5×10^5^ depicted as red color. **F,** Representative pictures of a subcutaneously grown tumour with a reached finalization endpoint of 1.5 cm diameter (*in vivo*). **G,** Kaplan–Meier plot showing survival of tumour mice (differences in median survival are indicated as Δ days). **H,** Cx3cl1 expression at mRNA level was measured in tumour tissue via qRT-PCR and normalised to the housekeeping control. **I,** Intratumoural cell proliferation was assessed via digital Ki67 marker IHC analysis, and positive cells were are given as percentage of total cells. **J-K,** Tumour-infiltrating CD3^+^ T-cells and CD8^+^ cytotoxic T-cells (**J**) and Foxp3^+^ regulatory T-cells (**K**) were determined immunohistochemically. DAB positive cells were manually counted in five report images. **L**, Intratumoural F4/80-positive macrophages were immunohistochemically stained and digitally analysed as percent of total cells. Horizontal lines in **H-L** indicate the mean, each dot indicates data from one individual mouse.

Having taken the adaptive immune response out of the equation by using athymic nude mice, we next wanted to test the impact of the peritoneal milieu on the CX3CL1-mediated effects described above. To this end, we generated ID8*luc*-*Trp53-/-Cx3cl1^+^* cells by lentiviral transduction to allow for bioluminescence measurements. These cells expressed approximately twice as much Cx3cl1 than the respective control cells (Figg. 4A and S3), and did not differ in tumour cell proliferation *in vitro* (Fig. 4B). 1×10^7^ ID8*luc*-*Trp53^−/−^*Ctrl or *Cx3cl1^+^* cells were subcutaneously implanted into female C57BL/6 mice. After one month, palpable tumour growth was documented once per week via bioluminescence measurement. Over the further course, the mean diameter of ID8*luc*-*Trp53^−/−^*Ctrl tumours was significantly higher than in the *Cx3cl1^+^* group (*P <* 0.0001; Fig. 4C). Consistent with these data, the photon flux of implanted luciferase-expressing tumour cells was significantly stronger within the ID8*luc*-*Trp53^−/-^*Ctrl group after luciferin substrate injection (*P =* 0.008; Fig. 4D and E). Tumour implants reached defined endpoints by 1.5 cm tumour diameter or exulceration (Fig. 4F). Finally, in contrast to the results in the intraperitoneal model, Cx3cl1 led to a significantly reduced tumour growth in the primary tumour model. The median overall survival in the ID8*luc*-*Trp53^−/−^*Ctrl group was 146 days after tumour cell inoculation, compared to 184 days in the *Cx3cl1^+^* group (*P =* 0.0049; Fig. 4G). Compared to the ID8*luc*-*Trp53^-/-^*Ctrl group, *Cx3cl1* mRNA expression was still 1.5-fold higher in subcutaneous tumour tissues of the *Cx3cl1^+^* group, albeit not reaching statistical significance after that long time (1.84 vs. 1.26 2^-ΔCt, *P =* 0.27, Fig. 4H). Ki67 staining once again revealed equal intratumoural proliferation rates with 25 % Ki67-positive cells in the ID8*luc*-*Trp53^-/-^*Ctrl group and 27 % in the *Cx3cl1^+^* group (*P =* 0.72; Fig. 4I). Most importantly, the infiltration of CD3-positive T-cells was doubled by the *Cx3cl1* overexpression (median 26 vs. 13 cells/report image, *P =* 0.04; Fig. 4J). CD8-positive cytotoxic T-cells were approximately 1.6-fold more abundant in *Cx3cl1^+^* tumours (*P =* 0.029; Fig. 4J). The intratumoural numbers of Foxp3^+^ regulatory T-cells (Fig. 4K) or F4/80^+^ macrophages (45 % vs. 46 % of total cells, *P =* 0.91; Fig. 4L) were not different between the groups.

In summary, specific features of the peritoneal milieu appear to be responsible for surpassing the adaptive anti-tumour immune response and for the negative net effect of CX3CL1 overexpression on tumour spread and survival in ovarian cancer.

### PARP inhibitors induce CX3CL1 secretion from human ovarian cancer cells

In recent years PARP inhibition has emerged as a cornerstone in the maintenance treatment of advanced ovarian cancer, significantly improving outcome especially in, but not restricted to, ovarian cancers with a deficiency in homologous recombination, e.g. *BRCA* mutations [15]. Thereby, one mechanism of action is that PARP inhibitors activate the cGAS/STING pathway, which in turn activates the induction of T-cell recruiting chemokines such as CCL5 or CXCL10 [2]. This pathway is also activated upon *BRCA* loss-of-function, and a recent report identified upregulation of *CX3CL1* as a downstrem event of *BRCA* mutations as well [16]. We therefore tested, whether PARP inhibition as well leads to enhanced CX3CL1 expression *in vitro*.

**Fig. 5:**
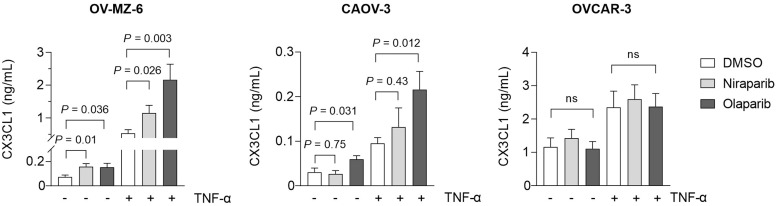
PARP inhibition enhances CX3CL1 secretion from human ovarian cancer cell lines. Human ovarian cancer cell lines OV-MZ-6, CAOV-3 and OVCAR-3 were stimulated with 10 µM olaparib, 10 µM niraparib or corresponding DMSO solvent control in the absence or presence of TNF-α (10 ng/mL). After 48 h, cell supernatants were harvested, and soluble CX3CL1 concentrations were assessed via ELISA.

Human ovarian cancer cell lines were stimulated with or without PARP inhibitors olaparib and niraparib in the presence or absence of TNF-α (Fig. 5). In OV-MZ-6 cells incubated for 48 h with 10 µM PARPi or solvent control (DMSO), CX3CL1 was 2.2-fold induced by niraparib (0.07 vs. 0.16 ng/mL, *P =* 0.011) and 2.1-fold by olaparib (0.07 vs. 0.15 ng/mL, *P =* 0.036). In the presence of TNF-α, the olaparib effect could be increased to 4.1-fold (0.53 vs. 2.16 ng/mL, *P =* 0.0031). In CAOV-3 cells only olaparib had a significant effect and doubled soluble CX3CL1 (0.03 vs. 0.06 ng/mL, *P =* 0.031; with TNF-α: 0.10 vs. 0.21 ng/mL, *P =* 0.0123). OVCAR-3 cells, which express the highest basal levels of CX3CL1 compared with the two other tested cell lines, could not be further elevated in CX3CL1 secretion under niraparib (1.16 vs. 1.42 ng/mL, p = 0.4957) or olaparib (1.16 vs. 1.11 ng/mL, p = 0.8812). TNF- α induced higher CX3CL1 levels, but again no differences could be detected if the control was compared to PARPi stimulations. The induction of CX3CL1 could therefore modulate the tumour-suppressive activity of PARP inhibitors in certain patients.

## Discussion

In the present work, we have identified CX3CL1 for the first time as a prognostic marker for poor survival in patients with advanced high-grade serous ovarian cancer, independent of other classical risk factors. This is in clear contradiction to corresponding studies in numerous other entities and preclinical studies that describe a tumour suppressive effect *in vivo* [[17], [18], [19], [20], [21], [22], [23]]. However, even in HGSOC itself, this distinguishes CX3CL1 from other T-cell recruiting chemokines such as CXCL9 and CXCL10, which can combat tumour growth *in vivo* by enhancing lymphocyte infiltration and represent a strong and robust protective prognostic marker [7,24,25]. Our *in vivo* studies also functionally confirm this negative impact, and one explanation may lie in the peculiarity of the peritoneal milieu in which ovarian cancer grows. Our studies show that the influence of CX3CL1 on tumour growth is highly dependent on the surrounding milieu.

As we expected, CX3CL1 enhanced T-cell recruitment, one of the general roles of this chemokine. And yet, CX3CL1 overexpression accelerated intraperitoneal tumour growth and shortened survival *in vivo*. These two opposing functions became evident in athymic nude mice, in which removal of the adaptive (T-cell triggered) immune response significantly augmented the CX3CL1-mediated survival disadvantage. One possible explanation for the accelerated intraperitoneal tumor growth would be an increased recruitment of tumor-associated macrophages, which has previously been implicated in peritoneal metastasis of ovarian cancer [26]. Indeed, we too observed increased macrophage infiltration in the i.p. model. However, (1) we see only a small proportion of tumor-promoting M2 macrophages, which remains unchanged by CX3CL1, (2) we fail to reproduce macrophage recruitment in athmic nude mice despite having significantly poorer survival, and (3) work by other groups showed that macrophage inhibition in the ID8 model tends to accelerate tumour growth, matching our observation of an increased macrophage infiltration by CX3CL1 [27]. This makes a contribution of macrophages to the observed CX3CL1 effects unlikely. Furthermore, we do not see an enhanced number of myeloid-derived suppressor cells. We also cannot confirm an increased proliferation of tumour cells by CX3CL1 overexpression, as has been demonstrated in the ovarian carcinoma cell line BG-1 [28], either in the growth curves *in vitro* or by proliferation markers such as Ki67 *in vivo*, and therefore it does not appear to be causative.

We therefore hypothesise that tumour cell-tumour cell or tumour cell-mesothelial cell interactions through the CX3CL1/CX3CR1 system may be responsible for the tumour-promoting effect. For the CX3CR1 receptor, previous work already indicated that it is able to mediate ovarian cancer cell chemotaxis and adhesion to peritoneal mesothelial cells [10,11]. We have reported similar findings for the CXCR3 chemokine receptor before [29]. Barbolina and colleagues were able to demonstrate that downregulation of CX3CR1 leads to reduced peritoneal metastasis, and increased receptor expression in human ovarian cancer was associated with a worsened prognosis [10]. However, CX3CR1 induction by CX3CL1 does not appear to participate in these processes (Fig. S2D). As we found CX3CR1 expression in the tumour cells of the mouse model as well as high levels of CX3CL1 in peritoneal metastases of the HGSOC cohort, we agree that the receptor may play an important role for the overall negative outcome in survival. Interestingly, we have observed significantly higher CX3CL1 expression in peritoneal compared to omental metastases in humans, an observation that Gurler Main et al. had also made in the corresponding tumour-affected mouse tissues in their work [10].

The fact that the promoting effect of CX3CL1 on tumour growth was completely reversed in the s.c. model despite comparable CX3CL1 overexpression further supports the special role of the peritoneal milieu. In the s.c. model, where we were able to blank the peritoneal environment out, we assume that CX3CL1-mediated immune activation slows tumour growth, as we and others have been able to show in other tumour models *in vivo* [14]. This is probably the case, because there is no possible adhesion site of membrane-bound CX3CL1 as presented for example at the aforementioned mesothelial cells of the peritoneum, and the CX3CR1-positive tumour cells instead remain adhesively attached within the primary tumour. Of course, the robustness of this model is limited to the abnormal composition of the subcutaneously grown tumour stroma, but nevertheless, we could find very comparable absolute amounts of immune cells and macrophages as seen in the intraperitoneal model.

Our observation that PARP inhibitors are able to induce CX3CL1 independently of the presence of inflammatory cytokines in ovarian cancer cell lines provides a first indication that this chemokine system can be modulated by contemporary therapies. The strength of induction *in vitro* was of the same order of magnitude as our forced overexpression *in vivo*, so that this mechanism may be functionally relevant in human ovarian cancer as well. It is likely that the PARP inhibitor-mediated activation of the cGAS/STING pathway is responsible for this CX3CL1 induction, as it has also been found in BRCA-deficient tumour cells, causing similar STING activation as PARP inhibitors [16]. Further studies should now elucidate these mechanisms *in vitro* and *in vivo*, as on the one hand they contribute to the already known improvement of immune infiltration by PARP inhibitors, but on the other hand they could also limit the therapeutic efficacy of these substances through the CX3CL1 net effect. In particular, a major focus here will be on the possibly different roles of soluble and membrane-bound CX3CL1. So far, the different roles of these two chemokine forms have only been taken into account in very few tumour models [30].

Taken together, we show in the present study that CX3CL1, despite activating the adaptive anti-tumour response in ovarian cancer, has an overall tumour-promoting function, which is due in particular to the peculiarities of the intraperitoneal milieu. It is now necessary to further clarify how this can be pharmacologically exploited for the benefit of improved tumour suppression.

## CRediT authorship contribution statement

**Stefanie Seitz:** Data curation, Formal analysis, Investigation, Methodology, Writing – original draft. **Tobias F. Dreyer:** Data curation, Formal analysis, Investigation, Methodology, Project administration, Supervision, Writing – review & editing. **Christoph Stange:** Data curation, Investigation. **Katja Steiger:** Investigation, Methodology. **Dirk Wohlleber:** Methodology. **Martina Anton:** Investigation, Methodology. **Thuý An Pham:** Investigation. **Dominique Sauter-Peschke:** Investigation. **Ute Reuning:** Data curation, Formal analysis, Resources, Supervision. **Gabriele Multhoff:** Investigation, Methodology, Project administration, Resources. **Wilko Weichert:** Methodology, Project administration, Supervision. **Marion Kiechle:** Methodology, Project administration, Supervision. **Viktor Magdolen:** Data curation, Formal analysis, Funding acquisition, Investigation, Methodology, Project administration, Supervision, Writing – review & editing. **Holger Bronger:** Conceptualization, Data curation, Formal analysis, Funding acquisition, Investigation, Methodology, Project administration, Resources, Supervision, Validation, Visualization, Writing – original draft.

## Declaration of competing interest

The authors declare the following financial interests/personal relationships which may be considered as potential competing interests:

KS receives research funding from Roche and is member of the advisory board of TRIMT GmbH. She also has filed a patent on a radiopharmaceutical compound. WW was as a member of advisory boards and speaker for Roche, MSD, BMS, AstraZeneca, Pfizer, Merck, Lilly, Boehringer, Novartis, Takeda, Bayer, Amgen, Astellas, Eisai, Illumina, Siemens, Agilent, ADC, GSK and Molecular Health, and he reports research funding from Roche, MSD, BMS and AstraZeneca, all outside of the submitted work. MK reports renumerations von Springer Press, Biermann Press, Celgene, Astra Zeneca, Myriad Genetics, TEVA, Eli Lilly, GSK, consulting for Myriad Genetics, Bavarian KVB, DKMS Life, BLAEK, TEVA, Exeltis, equity ownership in Therawis Diagnostic GmbH, AIM GmbH, funding from Sphingotec, Deutsche Krebshilfe, DFG, Senator Roesner Foundation, Dr. Pommer-Jung Foundation, Waltraut Bergmann Foundation, Bavarian State Ministry of Economy, BMBF. VM reports equity ownership in Therawis Diagnostic GmbH and a non-project-related grant from this company. HB reports grants from Deutsche Forschungsgemeinschaft (DFG), German Research Foundation during the conduct of the study, as well as personal fees from Roche, AstraZeneca, and GlaxoSmithKline outside the submitted work. No disclosures were reported by the other authors.

## Appendix Supplementary materials

Image, application 1
