# Supplementary material for: The chemokine CX3CL1 promotes intraperitoneal tumour growth despite enhanced T-cell recruitment in ovarian cancer
Source: Neoplasia. 2025 Jan 24;60:101130. doi: 10.1016/j.neo.2025.101130 (PMC11804824; doi:10.1016/j.neo.2025.101130)
Supplement: Supplementary file 1 [file mmc1.docx]

**Supplementary Materials and Methods**

**FACS Analysis.** In order to address changes in the presence of membrane-bound human or murine CX3CL1, human cell lines OV-MZ-6, OVCAR-3 or murine cell line ID8-*Trp53^-/-^* were stimulated for 24 h with or without TNF-α (50 ng/mL) for chemokine upregulation as well as with or without TAPI-2 (50 µM) for ADAM17 inhibition. Hereafter, cells were detached and 500,000 cells per sample were transferred in a 96-well V-shape plate. Between incubation steps, cells were centrifugated and washed with 100 µL FACS-buffer (0.5% FCS and 2 mM EDTA in PBS). Human cells were stained for 2 h on ice with 200 µg/mL human CX3CL1 polyclonal goat IgG primary antibody (#AF365, R&D Systems) or 200 µg/mL isotype polyclonal goat IgG (#AB-108-C, R&D Systems). After washing, 2 µL of Alexa Fluor 488 rabbit anti-goat IgG secondary antibody (#A-11078, Thermo Fisher Scientific) was added to the cells (30 min on ice in the dark). Murine cells were firstly treated with a mouse Fc-block (1:300, #553142, BD Biosciences), before 2 µg/mL Alexa-Fluor 488 conjugated monoclonal rat anti-mCX3CL1 (clone 126315, #FAB571G-025, R&D Systems) or respective isotype control (clone 54447, #IC006G, R&D Systems) was applied for 1 h in the dark. After the final centrifugation, human or murine cells were resuspended in 175 µL FACS-buffer and 1 µL 7-AAD Viability Staining Solution (#00-6993-50, eBioscience) to be measured as 20,000 events with Becton Dickinson FACS Calibur and CellQuest Pro Software (FL-1 filter 530 nm, FL-3 filter 670 longpass). 7AAD-stained dead cells as well as doublets were excluded in the final overlay histograms and dot plots.

**Quantitative reverse transcriptase PCR.** RNA from cell pellets or granulated tumour tissue was extracted by the RNeasy^®^ Plus Mini Kit (#74134, Qiagen) following the manufacturer’s protocol. cDNA was synthesised with 1 µg RNA via SuperScript™ IV First-Strand Synthesis System (#18091050, Thermo Fisher Scientific) and subsequently amplified using Brilliant III Ultra-Fast QPCR Master Mix (#600890-51, Agilent Technologies) and Thermo Fisher probes including primers (#Mm00436454_m1Cx3cl1 and #Mm03024075_m1Hprt). Data was generated using the MxPro qPCR Software. Gene expression levels were normalised to Hprt housekeeping gene and quantification was performed using the ΔCt calculation method.

**Cell proliferation analysis.** Proliferation of ID8-*Trp53^-/-^*Control and *Cx3cl1^+^* cells was compared due to metabolic activity by MTT assay at 4, 24 and 48 h. Briefly, cells were seeded at 2,000 cells per well in 96-well plate triplicates in DMEM supplemented with 5% FCS. MTT reagent was added for 3 h at 37 °C, cells were lysed with 150 µL DMSO and optical density was assessed at 570 nm in a microplate reader. ID8*luc*-*Trp53^-/-^*Control and *Cx3cl1^+^* cells were manually counted with a Neubauer counting chamber at time points 4, 24 and 48 h after 50,000 cells were seeded in duplicates in 12-well plates.

**Enzyme-linked immunosorbent assay (ELISA).** To evaluate Cx3cl1 concentrations in murine ascites, the fluid was centrifugated and 100 µl of the supernatant was used for antigen determination applying mouse CX3CL/Fractalkine DuoSet ELISA Kit from R&D Systems (#DY472, R&D Systems). The resulting chemokine concentration was normalised to the total protein amount of ascitic fluid by performing a Bradford assay with Coomassie Briliant Blue G-250 (#27815, Sigma-Aldrich) and bovine serum albumin (#1470, Sigma-Aldrich). For *in vitro* experiments, 100 µl cell supernatants were assessed for soluble chemokine concentrations with the human or mouse CX3CL1 DuoSet ELISA Kit (#DY365 or #DY472, R&D Systems) as well as the human or mouse CXCL10 DuoSet ELISA Kit (#DY266 or #DY466, R&D Systems).


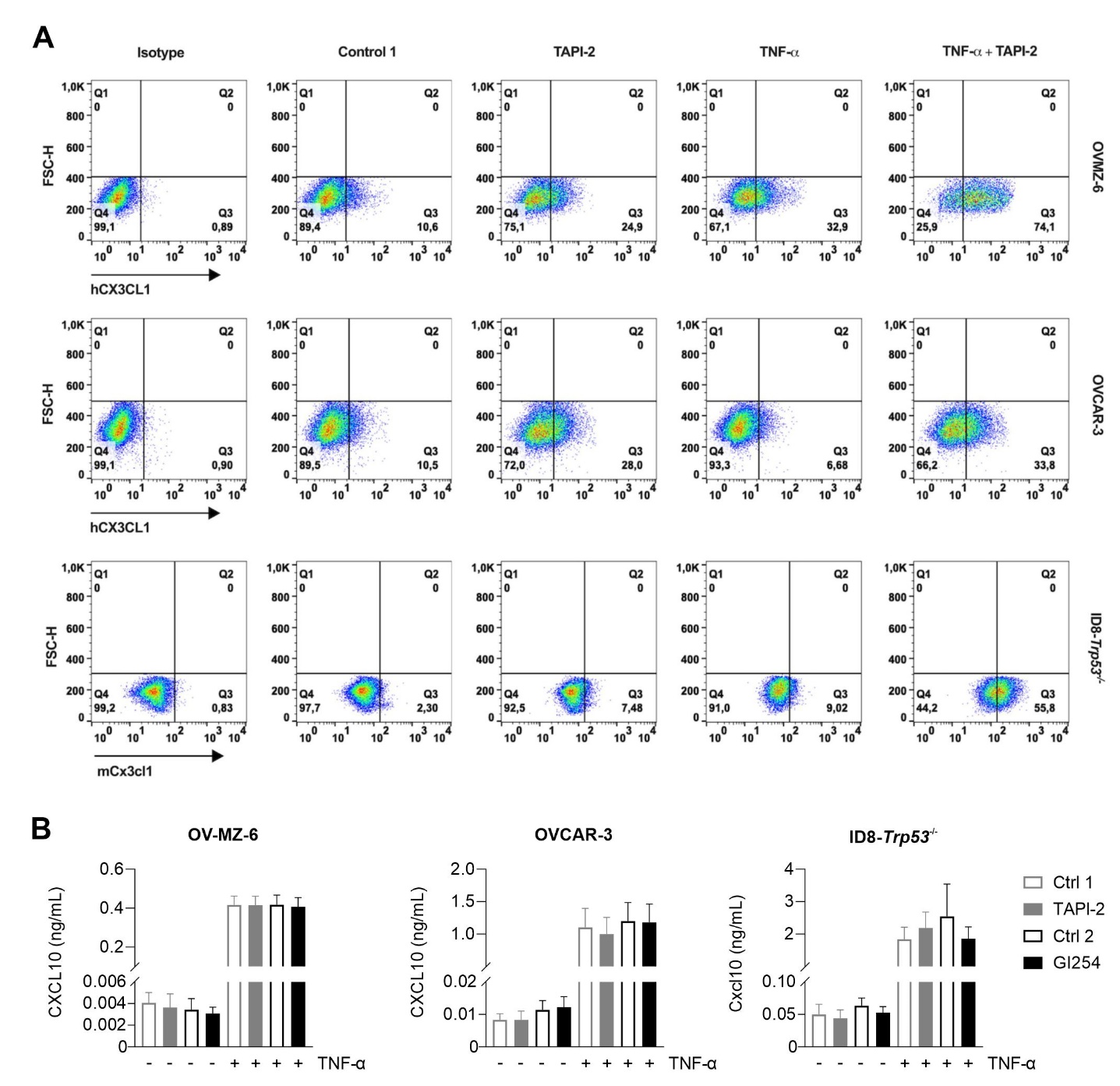


**Figure S1: A,** Dot plots represent the amount of membrane-bound CX3CL1 fluorescence on OV‑MZ‑6, OVCAR‑3 and ID8‑*Trp53^‑/‑^* cells under ADAM‑10 or ADAM‑17 inhibition, which was determined by FACS analysis. **B,** OV‑MZ‑6 cells, OVCAR‑3 cells, or ID8‑*Trp53^‑/‑^* cells were stimulated for 24 h ± TNF‑α (50 ng/mL) and ± TAPI‑2 (50 µM) or ± GI254 (5 µM) or the respective solvent controls. Concentrations of soluble human or murine CXCL10 in supernatants were assessed via ELISA and shown as mean ± SEM.


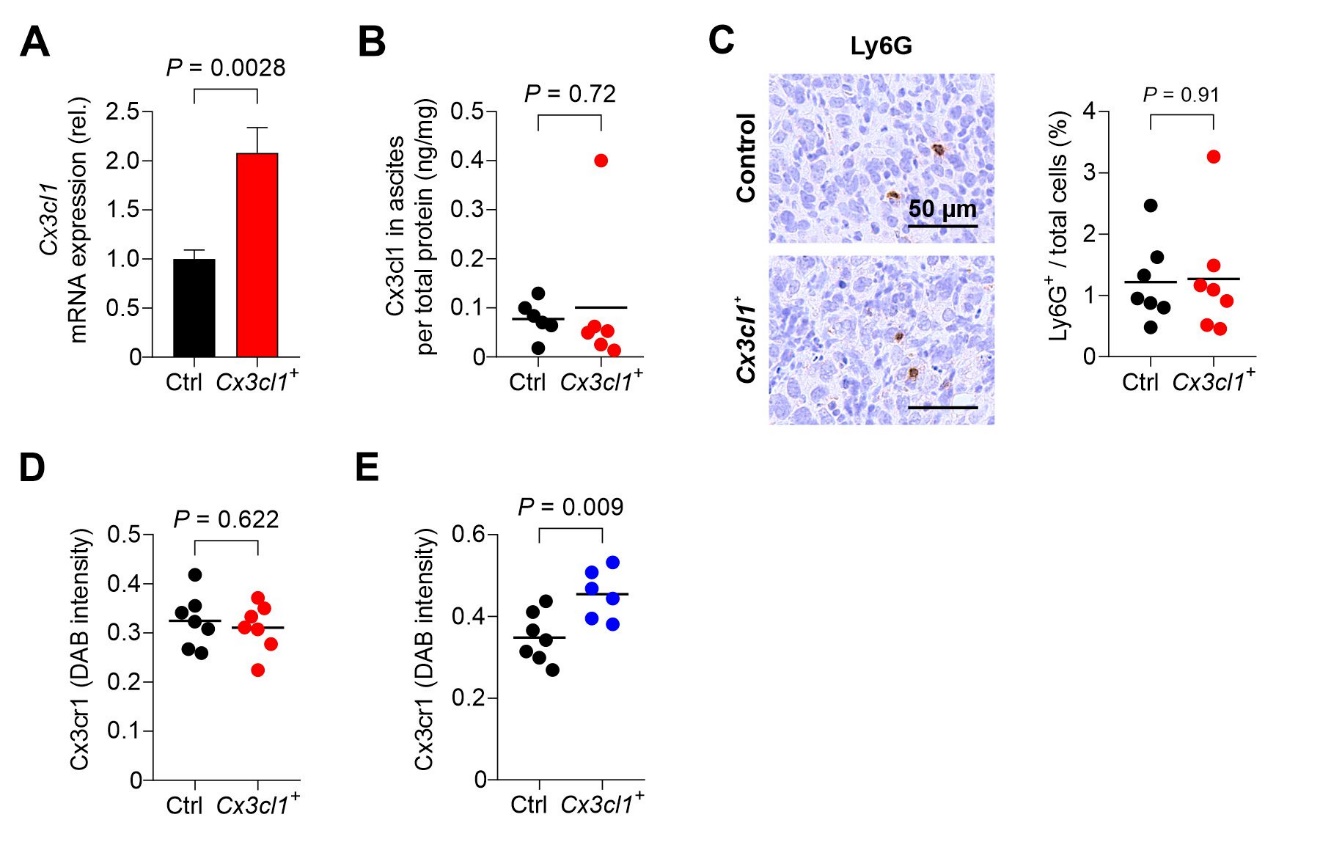


**Figure S2: A,** Relative *Cx3cl1* mRNA expression compared between ID8‑*Trp53^‑/‑^*Ctrl or *Cx3cl1^+^* cells. **B,** Soluble Cx3cl1 in ascites of C57BL/6 mice (see experiment Fig. 3D) measured via ELISA and normalised to total protein via Bradford assay. **C,** Digitally analysed immunohistochemical staining of intratumoural myeloid derived suppressor cells (Ly6G^+^) in ID8‑*Trp53^‑/‑^*Ctrl or *Cx3cl1^+^* tumours from C57BL/6 mice. **D‑E,** Ubiquitous Cx3cr1 staining was digitally compared between groups with DAB intensity values for the tumours derived from C57BL/6 (**D**) or athymic nude mice (**E**), respectively. Horizontal lines in **B‑E** represent the mean, dots indicate data from one individual mouse.


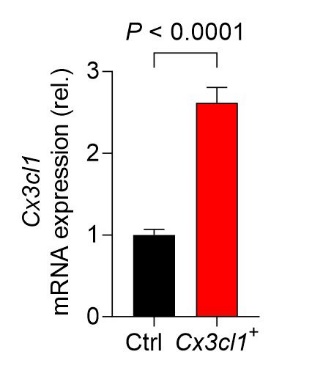


**Figure S3:** *Cx3cl1* mRNA expression was compared between ID8*luc*‑*Trp53^‑/‑^*Ctrl or *Cx3cl1^+^* cells via qPCR. Bars represent mean ± SEM.

**Table S1.** Patient demographics and clinicopathologic characteristics of the high-grade serous ovarian cancer patient cohort (n = 219).

| Clinical parameters | |  |  | n | range or % |
| --- | --- | --- | --- | --- | --- |
|  |  |  |  |  |  |
| Median age at diagnosis (years) (range) | | | |  | 64 (33-88) |
| ≤ 60 at initial diagnosis | |  |  | 79 | (36.1) |
| > 60 at initial diagnosis | |  |  | 180 | (63.9) |
|  |  |  |  |  |  |
| Median follow up time PFS (months) (range) | | | | 15 | (1-86) |
| Median follow up time OS (months) (range) | | | | 34 | (1-269) |
|  |  |  |  |  |  |
| FIGO stage |  |  |  |  |  |
| III |  |  |  | 163 | (74.4) |
| IV |  |  |  | 56 | (25.6) |
|  |  |  |  |  |  |
| Postsurgical residual tumour mass | | |  |  |  |
| 0 cm |  |  |  | 89 | (40.6) |
| ≤ 1 cm |  |  |  | 63 | (28.8) |
| > 1 cm |  |  |  | 63 | (28.8) |
| No data available | |  |  | 4 | (1.8) |
|  |  |  |  |  |  |
| Ascites volume | |  |  |  |  |
| 0 ml |  |  |  | 25 | (11.4) |
| ≤ 500 mL |  |  |  | 35 | (16.0) |
| > 500 mL |  |  |  | 57 | (26.0) |
| No data available | |  |  | 102 | (46.6) |
|  |  |  |  |  |  |
| Nodal status | |  |  |  |  |
| Negative (pN0) | |  |  | 57 | (26.0) |
| Positive (pN1) | |  |  | 128 | (58.4) |
| No data available | |  |  | 34 | (15.5) |
|  | |  |  |  |  |

**FIGO**, International Federation of Gynecology and Obstetrics; **OS**, overall survival; **PFS**, progression-free survival.

**Table S2.** Univariate Cox regression analysis of clinical outcome in high-grade serous ovarian cancer patients (FIGO III/IV) with respect to clinical parameters and CX3CL1 expression.

| **Clinical parameters** | **n** | **PFS** | ***P*** | **n** | **OS** | ***P*** |
| --- | --- | --- | --- | --- | --- | --- |
|  |  | **HR (95% CI)** |  |  | **HR (95% CI)** |  |
|  |  |  |  |  |  |  |
| **Age (years)** |  |  | 0.303 |  |  | 0.851 |
| ≤ 60 | 71 | 1 |  | 79 | 1 |  |
| > 60 | 119 | 1.19 (0.85-1.67) |  | 140 | 1.03 (0.74-1.44) |  |
|  |  |  |  |  |  |  |
| **FIGO stage** |  |  | **0.004** |  |  | **0.001** |
| III | 146 | 1 |  | 163 | 1 |  |
| IV | 44 | 1.76 (1.20-2.57) |  | 56 | 1.77 (1.25-2.51) |  |
|  |  |  |  |  |  |  |
| **Residual tumour mass** |  |  | **<0.001** |  |  | **<0.001** |
| 0 cm | 81 | 1 |  | 89 | 1 |  |
| > 0 cm | 106 | 2.04 (1.47-2.84) |  | 126 | 3.06 (2.13-4.38) |  |
|  |  |  |  |  |  |  |
| **Ascites volume** |  |  | **<0.001** |  |  | **<0.001** |
| ≤ 500 mL | 54 | 1 |  | 60 | 1 |  |
| > 500 mL | 50 | 2.49 (1.59-3.90) |  | 57 | 2.16 (1.44-3.24) |  |
|  |  |  |  |  |  |  |
| **Nodal status** |  |  | 0.216 |  |  | **0.010** |
| negative | 53 | 1 |  | 57 | 1 |  |
| positive | 110 | 1.27 (0.87-1.84) |  | 128 | 1.70 (1.13-2.54) |  |
|  |  |  |  |  |  |  |
| **CX3CL1 expression** |  |  | **0.026** |  |  | **0.018** |
| low | 112 | 1 |  | 128 | 1 |  |
| high | 78 | 1.44 (1.04-2.00) |  | 91 | 1.47 (1.07-2.03) |  |
|  |  |  |  |  |  |  |

**CI**, confidence interval; **FIGO**, International Federation of Gynecology and Obstetrics; **HR**, hazard ratio; **OS**, overall survival; **PFS**, progression-free survival. Significant values (*P*< 0.05) are indicated in bold.
